# The Molecular Genetics of Marfan Syndrome

**DOI:** 10.7150/ijms.60685

**Published:** 2021-05-27

**Authors:** Qiu Du, Dingding Zhang, Yue Zhuang, Qiongrong Xia, Taishen Wen, Haiping Jia

**Affiliations:** 1 Marfan Research Group, College of Medical Technology, Chengdu University of Traditional Chinese Medicine, Chengdu, 610072, Sichuan, China.; 2 Sichuan Provincial Key Laboratory for Genetic Disease, Sichuan Provincial People's Hospital, School of Medicine, University of Electronic Science and Technology of China, Chengdu, 611731, Sichuan, China.; 3 Department of Rheumatology and Immunology, Sichuan Provincial People's Hospital, University of Electronic Science and Technology of China, Chengdu, 611731, Sichuan, China.; 4 Department of Immunology, North Sichuan Medical College, Nanchong, 637100, Sichuan, China.

**Keywords:** Marfan syndrome, diagnose, genetics, technology

## Abstract

Marfan syndrome (MFS) is a complex connective tissue disease that is primarily characterized by cardiovascular, ocular and skeletal systems disorders. Despite its rarity, MFS severely impacts the quality of life of the patients. It has been shown that molecular genetic factors serve critical roles in the pathogenesis of MFS.* FBN1* is associated with MFS and the other genes such as *FBN2*, transforming growth factor beta (TGF-β) receptors (*TGFBR1* and *TGFBR2*), latent TGF-β-binding protein 2 (*LTBP2*) and *SKI*, amongst others also have their associated syndromes, however high overlap may exist between these syndromes and MFS. Abnormalities in the TGF-β signaling pathway also contribute to the development of aneurysms in patients with MFS, although the detailed molecular mechanism remains unclear. Mutant FBN1 protein may cause unstableness in elastic structures, thereby perturbing the TGF-β signaling pathway, which regulates several processes in cells. Additionally, DNA methylation of *FBN1* and histone acetylation in an MFS mouse model demonstrated that epigenetic factors play a regulatory role in MFS. The purpose of the present review is to provide an up-to-date understanding of MFS-related genes and relevant assessment technologies, with the aim of laying a foundation for the early diagnosis, consultation and treatment of MFS.

## Introduction

Marfan syndrome (MFS), a complicated genetic connective tissue disorder named after Antoine-Bernard Marfan in 1896, presents with striking pleiotropism and clinical variability. Patients with MFS exhibit a wide range of clinical symptoms, including abnormalities in the ocular, skeletal and cardiovascular systems. Aortic pathology is the predominant cause of death in patients with MFS 1. Mild cases may only have isolated MFS characteristics, whereas severe cases have rapidly progressive lesions involving multiple systems during the neonatal period, and may succumb to the disease within 2 years after birth 2,3. MFS affects ~1/5,000 individuals in the general population 4. The prevalence of MFS in the Danish population in 2015 was 6.5/100,000, which was 41% higher compared with 20 years ago 5. The average lifespan of Danish patients with MFS was 50 years 6. The average lifespan of MFS patients was 32 years in 1970s. With the development of aortic root replacement treatment, the mean lifespan expectancy of MFS increased to 41 years old in 1995 7, even nearly 72 years old with proper management 8. Notably, Veiga-Fernández *et al* showed that 34.54% of early-onset MFS (EOMS) cases were suspected based on prenatal ultrasound anomalies, and 65.45% of EOMS cases were diagnosed after birth. The mortality rate in the first 15 months was 73.68% in cases with prenatal suspicion, and 61.1% of patients that were postnatally diagnosed died within the first 5 years 3. Of note, MFS not only has a high mortality rate among newborns, but also seriously affects the quality of life of surviving patients.

Although sporadic cases of MFS account for 25-30% of those diagnosed 9, relevant clinical data provide evidence that MFS is primarily an autosomal dominant disease. Additionally, there are a few cases of MFS families with a reported autosomal recessive inheritance model 10, 11. Several patients with MFS can be traced based on their family history. Manifestations of MFS may worsen with age, although early-stage symptoms are not apparent/visible. For mild cases, early-stage diagnosis may be delayed due to a lack of symptoms. Certain manifestations of MFS also overlap with other diseases, such as Loeys-Dietz syndrome (LDS) and Shprintzen-Goldberg syndrome (SGS) 12. Additionally, the molecular pathogenesis of MFS has not been fully established.

Aortic development in patients with MFS presents sexual dimorphism. Aortic aneurysms in patients with MFS are more severe in males compared with females 13. Early diagnosis of MFS is useful in preventing cardiovascular symptoms and may encourage early treatment of the disease, thereby improving the quality of life of the patients and potentially prolonging their lifespan. Diagnostic criteria involving multiple system manifestation scores and updated diagnostic criteria in 2010 emphasized the importance of fibrillin-1 (*FBN1*) genetic testing 14. Currently, specific gene mutations, including *FBN2*, transforming growth factor beta (TGF-β) receptors *(TGFBR1* and *TGFBR2*), latent TGFβ-binding protein 2 (*LTBP2*) and *SKI*, have been identified in their associated syndromes by several molecular genetic techniques, including linkage analysis, scanning methods and specific identification techniques. The present review focuses on the genetic aspects of MFS and the screening of MFS-associated genes.

## Clinical Characteristics of MFS

MFS involves abnormalities of multiple systems, including the cardiovascular, ocular and skeletal systems, and include lung, skin and central nervous system disorders 15. Patients with MFS may also develop sleep disorders and depressive symptoms due to long-term severe physical pain 16. Compared to the abnormalities of the cardiovascular system in patients with MFS, loss of vision or skeletal anomalies are more likely to be discovered earlier.

### Effects on the cardiovascular system

Pathological changes to the cardiovascular system are common amongst patients with MFS. The typical clinical manifestation of MFS is dilation of the aortic root, and the atrioventricular valves are the most likely affected tissues. The thickening of atrioventricular valves is often associated with atrioventricular valves prolapse 17. Rybczynski *et al* reported that the incidence of mitral valve prolapse (MVP), regurgitation and endocarditis was 42.6, 56.5 and 0.92% among 204 patients with MFS at 30 years of age 18. Furthermore, patients with MFS who appeared to be asymptomatic during childhood and adolescence, particularly female patients, gradually developed mitral valve dysfunction and aortic abnormalities at a constant rate between the ages of 5 and 20 years 19. The dysfunction of mitral valve causes left ventricle volume overload then evolving towards systolic and diastolic dysfunction of left ventricle 20. Moreover, in children with severe or EOMS, abnormal mitral valve function may cause regurgitation and lead to congestive heart failure, pulmonary hypertension and even death 21.

Aortic tears or ruptures due to continuous enlargement of the aorta were the primary causes of death. Dilation often occurs in the first part of the ascending aorta and progresses to an aortic aneurysm, which then forms a type A aortic dissection 22. Hascoet *et al* found that children who exhibited a Valsalva sinus Z-score increase of ≥0.1 per year or a Valsalva sinus Z-score ≥3 before the age of 16 years, had a higher risk of cardiovascular events 23. The prevalence of ascending aorta dilation increases with age, and ~96% of 965 patients with MFS developed ascending aorta dilation by the age of 60 years 24. The prevalence of aortic root dilatation, aortic valve regurgitation and MVP was similar amongst children and adults, whereas mitral valve regurgitation, pulmonary artery dilatation, aneurysms of the aortic arch, descending thoracic aorta and abdominal aorta were found predominantly in adults 25. Moreover, MFS have a high risk of recurrent aortic dissection, and the descending aortic size of patients with recurrent aortic dissection is greater than those with initial aortic dissection 26. MFS patients who receive the prophylactic aortic surgery have a substantial risk of type B aortic dissection 27. Dissection or aneurysm of the thoracic aorta was associated with degradation of support structures of the aortic wall, spine tortuosity and disturbed luminal blood flow caused by the tortuousness of the aorta 28. Jondeau *et al* reported that the risk of death or aortic dissection was associated with a 4-fold increase at the aortic diameter ≥50 mm, which was considered a threshold for surgery 29. European Society of Cardiology (ESC) recommended that surgical indications in MFS patients should be with maximal aortic diameter ≥50 mm, or MFS patients with maximal aortic diameter ≥45 mm and additional risk factors (e.g.family history of aortic dissection at a low diameter, progressive aortic regurgitation, desire for pregnancy, aortic diameter increase >3 mm/year) 30.

Welby *et al* confirmed that the rate of carotid artery tortuosity was 88% in patients with MFS and an increased prevalence of retrojugular course was associated with increased occurrence of aortic dissection 31. Moreover, visceral arterial tortuosity, the aortic tortuosity index (ATI) and vertebral tortuosity index (VTI) were found to be significantly increased in patients with MFS. ATI indicates the risk of severe aortic phenotypes that are classed as type B aortic dissection as well as the aortic volume expansion rate, whereas VTI is related to early-onset dissection and death. Both are potential markers of aortic involvement 32-34.

### Ophthalmological effects

Ocular defects, another major pathological manifestation of MFS, may precede cardiovascular system symptoms. Approximately 50-80% of patients with MFS exhibit ectopia lentis (EL). Compared with non-patients with MFS, additional manifestations amongst patients with MFS include an elongated axial length of the eyes, reduced visual acuity, flatter corneas, greater corneal astigmatism, a thinner central cornea, lower intraocular pressure, iris transillumination defects and lower values of sagittal height in the central cornea, corneal periphery and the sclera 35, 36. Wang *et al* suggested that the mean keratometry combined with central corneal thickness can be considered as a screening index for MFS 37. Patients with MFS are also at high risk of developing cataracts 38. Iris irradiance defects, EL and myopia are characteristic manifestations in childhood and adolescent MFS 39. The incidence of EL during the early stages of MFS varies widely, from 63-75% for children aged <10 years, to 15-57% for children aged <17 years 38. Therefore, ophthalmic testing should be performed in patients with MFS even during childhood, before any cardiovascular manifestations develop.

### Skeletal effects

It is generally hypothesized that the most common skeletal feature of patients with MFS is elongated bones, which may be characterized by a ratio of arm span to stature of >1.05. The overgrowth of ribs leads to pectus carinatum or pectus excavatum, and long finger and loose joints cause the wrist sign 17. Arachnodactyly and the thumb sign are typical symptoms. Pes planus is caused by the increased ligamentous laxity and varies from mild to severe deformity 17, 40. The scoliosis of a curve of 30°or greater is typical in MFS patients and more than 50°is severe, and the thoracic curve is usually convex to the right 40. The other skeletal symptoms include dural ectasia, acetabular protrusion, decreased bone mineral density and etc. 40. Sponseller et al found that ~27% of patients with MFS had skeletal lesions, 19% had <1 skeletal manifestation, and the common physical features amongst the patients were craniofacial characteristics, thumb and wrist signs, pectus excavatum and severe hindfoot valgus 41. There is no difference in the imaging characteristics of adolescents between MFS and idiopathic scoliosis, however, scoliosis-like symptoms in patients with MFS progresses more rapidly. The incidence of cerebrospinal fluid leakage and dual expansion in patients with MFS are higher compared with non-patients 42, 43. Thumb and wrist signs are common in children with MFS. Symptoms that involve the upper and lower limb extremities in patients with MFS typically appear first. Chest and spinal deformities may be less frequent in adolescents. The occurrence of pectus deformities, wrist signs and scoliosis as a result of MFS increases with age, whereas the prevalence of hypermobility and pes planus decreases with age 44, 45. Moreover, total body bone mineral content and muscle mass worsen with age in patients with MFS 46.

### Diagnostic criteria

Diagnostic criteria of MFS primarily depend on pathological manifestations of multiple systems and family history. Cardiovascular symptoms of MFS are emphasized in the revised Ghent criteria. In cases without a family history of MFS, patients with aortic root dissection or dilatation (Z-score ≥2) and one of the three MFS-related manifestations (EL, FBN1 mutation, the system score ≥7) can be diagnosed with MFS, and MFS diagnosis is also confirmed by the presence of EL and FBN1 mutation associated with aortic disease. Moreover, in cases with a family history of MFS, the presence of EL, or the systemic score ≥7 points or aortic root dilatation (Z-score ≥2 in individuals above 20 years old or Z-score ≥3 in individuals below 20 years old) are sufficient for an MFS diagnosis 14. In addition, when clinical manifestations do not meet the diagnostic criterion, gene mutation detection is a suitable means of early diagnosis 47.

The prevalence and average age of MFS diagnosis are increasing 5. This can be attributed to several factors: First, patients with MFS symptoms gradually worsen and do not reach the diagnostic criteria until an older age; thus, more people are now being diagnosed, although the actual true incidence may not have increased substantially; the mortality of MFS decreases with timely treatment, and the increasing number of patients with MFS is partially caused by a decrease in the relative risk of death; an increase in awareness of MFS has enabled the diagnosis of several cases in the adult population; finally, patients who live longer may increase the possibility of the transmission of mutants 5.

## Detection of Disease-associated Genes in MFS

Multiple gene loci related to MFS have been discovered through linkage analysis and other techniques, making the study of molecular genetic mechanisms underlying the development of MFS possible. In addition, detection of specific sequences reveals potential pathogenic mutations, and can advance diagnosis and targeted therapy for patients with MFS. Detection of *FBN1* mutation has been included in the revised Ghent criteria, before which only 56% of MFS children could be diagnosed without an* FBN1* mutation test, whereas 85% of children currently meet the criteria following molecular testing 48. These technologies are further discussed below.

### Linkage analysis for loci exploration

Linkage analysis focuses on pedigrees and is primarily used for single-gene diseases 49. Detection rates with linkage analysis depend on the heterogeneity of the disease locus, and may be impacted by incomplete penetrance instead of allele heterogeneity within one gene 49. Linkage analysis has been used to explore the potential pathological loci in MFS pedigrees. In 1992, Tsipouras *et al* mapped the single fibrillin locus of MFS on chromosome 15 in 28 families by genetic linkage analysis 50. In 1994, Collod *et al* identified a second locus of MFS located at 3p24.2-p25 by linkage analysis in a large French family without EL 51. The usage of linkage analysis in humans is limited within a finite family size and long generational times 52.

### Scanning methods for related genetic change detection

#### Single-strand conformation polymorphism (SSCP) analysis

The SSCP process includes PCR amplification of the target fragment. In the subsequent electrophoretic process, mutant and wild-type single strands fold into 3D shapes, showing various electrophoretic mobilities and bands in a non-denaturing gel 53. Since the mutation contains 60% G+C of 100-300 bp, it is easily detected, but the sensitivity of SSCP is affected by multiple factors, such as the gel matrix used, size of the DNA fragments and temperature setting during electrophoresis, amongst other factors 53, 54. Thus, there is the limitation of the size of the fragments in SSCP. Wang *et al* detected a 13 bp deletion (gccTc Tgcaccca) located on exon 25 of *FBN1* in 9 MFS families by SSCP using direct sequencing technology 55. Rommel *et al* found 7 missense mutations, 3 splice site alterations and 1 indel mutation of *FBN1* in 76 patients with MFS or related symptoms by SSCP and sequencing 56.

#### Conformation sensitive gel electrophoresis (CSGE)

CSGE is used to scan PCR amplification products. Conformational changes of products caused by single-base mismatches are amplified by a denaturing solvent and hence increase the migration difference of single-base mismatched double strands 57. CSGE is widely used in mutation screening of genetic diseases and detection of single-nucleotide polymorphisms, but can only detect single base changes, small insertions and deletions, with an optimal length of 200-500 bp 58. The detection rates of *FBN1* mutations by CSGE is >90% 59. Loeys *et al* performed an initial mutation analysis on the cDNA and gDNA of 93 patients with MFS by CSGE and SSCP analysis. In this analysis, 73 mutations were identified, amongst which, 52 were present in the gDNA and 21 were present in the cDNA 60. Uyeda *et al* reported 3 mutations of *FBN1* (c.719 C>T, c.4229 T>C and c.8121 G>C), and 10 *FBN3* single nucleotide polymorphisms in 12 Japanese patients with MFS by CSGE and direct sequencing 61.

#### Denaturing high-performance liquid chromatography (DHPLC)

In the presence of mutations, the mutant single strand and the wild-type single strand form a heteroduplex, and complementary mutant single strands or two wild-type single strands form a homoduplex, each with a different residence time on a reverse phase chromatography column. DHPLC has a high detection sensitivity of 0.5%, the minimum detection amount of the total analyzed DNA 62. The combination of DHPLC and direct sequencing is fast and cost-effective, but cannot detect the overall deletion and duplication of the entire exon 63. Huang *et al* identified a small insertion mutation (4307insTCGT) and a missense mutation (5309G>A) of *FBN1* in 9 patients with MFS by DHPLC and sequencing 64. Kosaki *et al* designed an automated and economical 'COPPER plate' to simultaneously detect all exons of one gene by PCR and DHPLC and, subsequently, the COPPER plate was developed for the detection of *FBN1* 65.

### Specific techniques for identification of MFS-associated genes

#### Whole exome sequencing (WES)

WES is a type of high-throughput sequencing method that functions by capturing DNA from the exonic region of the whole genome. The rare genetic events and novel mutations that are disease-associated (particularly for monogenic diseases) and potential disease-causing mutations due to incomplete penetrance can be detected by WES. Thus, it is widely used to diagnostically evaluate a patient's genetic condition 66, 67. Moreover, WES can improve the detection rate of prenatal genetic abnormalities and more medical prediction information can be provided based on whether the mutation occurs in an important functional area of the gene 68. The success of WES depends on mutations being compassed in the captured portion of genome and the ability to identify the pathogenic variant among many thousands of new variants 69. Yang *et al* performed WES to provide a molecular diagnosis for 25% of 3,386 patients with suspected genetic conditions 66. LaDuca *et al* reported that 99.7% of 153,300 pathogenic variants were detectable by WES, and 98.6% of 93,062,298 pathogenic variants presented an adequate depth for potential detection 70. Aubart *et al* found five relevant variants (c.1286 G>C in *FBN1*, c.304 G>A in *SMAD3*, c.1588 C>T, c.329 T>C and c.3164 C>T in *COL4A1)* in 51 patients with severe MFS by WES 71. However, 5-10% of genes (low-quality sequences) are rarely covered in WES, and sequences with high CG content are not easily captured 72, 73. Unfortunately, the detection of differences in copy number variation (CNV), translocations, repeat expansions, and tandem repeat size are unavailable by WES 68.

#### Whole genomic sequencing (WGS)

WGS is used to detect variants across the whole genome with the most continuous coverage, hence detecting coding and non-coding region variants of nuclear and mitochondrial genomes and increasing the detection ability of CNV and the mapping of genome-wide dense homozygosity 74, 75. However, the sequencing read length of short-read WGS may result in incomplete coverage of some sequences with mappability <1, and raw-reads of long-read WGS have a high error rate, not to mention that the cost of WGS can be prohibitive 74. Moreover, due to the limitations of databases (uncomprehensive and conflicting data) and knowledge about genic effects, it produces great difficulties for interpreting all of these sequencing data 76. Benke *et al* found a 31,956 bp deletion of FBN1 by WGS in a female MFS patient, and the deletion was also confirmed by multiplex ligation-dependent probe amplification (MLPA) and Sanger sequencing 77. Yuan *et al* reported a new preimplantation genetic testing (PGT) method combined with WGS for de novo mutations, and a paternal MFS family with a *de novo* mutation (c.4952_4955delAATG) in *FBN1* successfully had a healthy newborn infant by PGT and chromosomal balanced translocations 78.

#### Targeted genome sequencing (TGS)

TGS is used to screen a panel of markers known with clinical relevance by selective enrichment of the genomic areas where comprise these markers 79, 80. Compared with WES and WGS, TGS not only has greater sequencing depth but also the reduced overall costs and data burden 79. Li *et al* used panel-based targeted next-generation sequencing to analyze the *FBN1*, *TGFBR1* and *TGFBR2* genes in 123 Chinese with MFS or related disease and found that 97 cases had at least one pathogenic mutation 81. Wei *et al* combined the targeted DNA-HiSeq and next-generation sequencing to design an array-based gene chip for the detection of the exons of 193 genes associated with 103 genetic diseases 82. However, TGS has low ability to detect structural rearrangements or CNV 76.

#### Multiplex ligation-dependent probe amplification (MLPA)

MLPA is used to detect the deletions or duplications of specific genes and the presence of abnormal DNA methylation in diseases 83. Contamination of genomic DNA samples by PCR may lead to a false-positive result of MLPA 84. Yang *et al* found five novel large deletions in *FBN1* in 5 patients with MFS by MLPA. One of the deletions encompassed exons 44-66 in *FBN1*, and others encompassed exons 43, 56, 54 and 50 85. Furtado *et al* reported two novel large deletions in 4 patients with MFS by MLPA, including an *FBN1* deletion which encompassed exons 1-5, and a 542 Kb deletion in chromosome 15 that spanned the entire *FBN1* gene and another 5 genes 86. Moreover, Li *et al* demonstrated that in-frame deletions between exons 24-53 were related to severe clinical phenotypes. Patients with mild MFS showed an exon 6 deletion, and the classic MFS was linked to deletions of exons 1-36 by MLPA analysis 87. These studies not only supplement the *FBN1* mutation data, but also highlight the importance of detecting large *FBN1* deletions in patients with MFS.

#### Single cell sequencing (SCS)

SCS technologies explore the gene expression heterogeneity between cells by analyzing the genetic information in a single cell. One of the most commonly used SCS methods is single-cell RNA sequencing (scRNA-seq), which is used to reveal the regulation between genes and track the trajectories of cell lineages during development 88. Pedroza *et al* found a cluster of modulated smooth muscle cells (SMCs) in aortic aneurysm tissue of an adult *FBN1*^C1041G/+^ mouse model by scRNA seq, and upregulated activity of TGF-β signaling, and expression of Kruppel-like factor 4 (Klf4) may be a potential upstream driver that promotes SMCs modulation 89.

### Genes contribute to MFS and related diseases

MFS and related disorders including LDS, SGS, MASS phenotype etc., display a significant overlap. These diseases and nonsyndromic aneurysmal syndromes are associated with the abnormal of TGF-β signaling 12. Multiple genes contribute to MFS and related diseases, such as the FBN-encoding genes *FBN1* and *FBN2*, and genes encoding signaling molecules of the TGF-β pathway, such as *TGFBR1/2*, *LTBPs* and *SKI*. Mutations of *FBN1* were observed in >90% of cases of MFS 90 (Table 1).

### FBN1 gene in MFS

*FBN1* is located on chromosome 15q21.1 and is comprised of 66 exons. It is transcribed into a 10-Kb mRNA, which is then translated into FBN1, a 2,871 amino acid long protein with a large number of cysteine repeats. FBN1 is an extracellular matrix (ECM) glycoprotein and a structural component of microfibers with a diameter of 10-12 nanometers, and contains 47 epidermal growth factor (EGF)-like modules, 7 TGF-β-binding protein-like domains and 43 EGF-like modules with calcium-binding (cb)EGF consensus sequences. FBN1 is an important component of elastic fibers in the elastic or inelastic connective tissues that provides support for the load-bearing structures and a scaffold for protein precipitation 91-93. However, in a homozygous and heterozygous mgΔ mutant mice model, it has been shown that the primary role of FBN1 is to maintain tissue homeostasis, not to assemble the elastic matrix 94.

A total of 3,077 mutations of *FBN1* in patients with MFS have been reported to date, including 2,499 (73.09%) point mutations and 51 (1.66%) large rearrangements. Missense mutations of *FBN1* account for 53-56.1% of cases, 33-36.8% of truncated variants, 7.1-13% of intronic variants and 1.8-2.9% of gross genomic rearrangements 85, 90. Mutations of *FBN1* occur across almost the entire gene, and there is no obvious aggregation area and periodicity. The repetitiveness of this mutation is ~12%. Exon mutations can be found in most of the exonic regions in patients with MFS, whereas exons 45 and 57 are underrepresented and exons 13, 26, 27, 28 and 43 are overrepresented 95. However, Groth *et al* reported that the total number of variants in patients with MFS (diagnosed by calculating the MFS phenotype score) accounted for only 35.8% of all registered variants 96. They suggested that certain databases contained incorrectly interpreted conclusions of variants, and thus should only be considered as a reference for seeking information regarding the specific mutation 97.

### Genes associated with the related disease

#### FBN2

*FBN2* is located on chromosome 5q23.3, and the encoded protein, FBN2, is a component of connective tissue microfibrils. It has been shown that the developmental expression of *FBN2* is earlier than that of *FBN1* 98. FBN2 is related to the formation of elastic fiber structures, whereas FBN1 primarily maintains the function of elastic structures. Therefore, the expression of *FBN2* is common in elastic tissues, and *FBN1* is dominant in stress and weight-bearing structures 98, 99. Gupta *et al* found a mutation site of *FBN2* in a female proband and her brother with congenital contractual arachnodactyly, and the proband also met the diagnostic criteria of MFS, with progressive dilatation of the aorta at the sinuses of Valsalva, however, none of them presented with MVP or regurgitation 100.

#### TGFBR2

*TGFBR2* is located on chromosome 3p24.1. *TGFBR2* is associated with MFS at the second locus for MFS 101. *TGFBR2* encodes the TGFBR2 protein, which forms a complex with the TGFBR1 and binds to TGF-β mediating protein phosphorylation, and modulating cell proliferation, cell cycle progression and ECM formation 101. Mutations of *TGFBR2* are associated with MFS without major ocular symptoms 102. In nematode models, mutations of *TGFBR2* that cause MFS or MFS-like syndromes may disrupt the structure of TGFBR2 with an exposed surface domain, alter subcellular localization patterns, and indirectly alter the trafficking of the TGFBR1 103. Zhang *et al* found a p.V453E mutation of *TGFBR2* (located in the F-helix in the kinase domain) in a Chinese patient with MFS and two relatives with certain MFS-like manifestations. They demonstrated that mutations of *TGFBR2* located on the F-helix in the kinase domain may be related to severe cardiovascular and skeletal symptoms and minor ocular symptoms 104. Attias *et al* reported the cardiovascular symptoms of patients with MFS caused by *TGFBR2* mutations were similar in age and incidence of aortic dilatation to those of patients with *FBN1* mutations, and their therapeutic effects were similar. Therefore, the severity of MFS cannot be only attributed to the presence of *TGFBR2* mutations 105.

#### TGFBR1

*TGFBR1*, located on chromosome 9q22.33, consists of 9 exons and encodes the TGFBR1 protein. Mutations of *TGFBR1* may also be found in patients with MFS 106. Lucarini *et al* suggested that the 6Ala allele of *TGFBR1* may be considered as a low penetrance allele in patients with MFS 107. However, Somers *et al* demonstrated that 5 MFS patients with the TFGBR1*6A allele did not present phenotypic differences when compared with 21 MFS patients without a TFGBR1*6A allele, although this may partly be due to the small sample size of the study 108.

*TGFBRs* is also considered a causative gene of LDS.Certain clinical manifestations of LDS and MFS are similar, including cardiovascular symptoms, scoliosis and craniofacial features. Hence, it may be difficult to evaluate the genetic role of *TGFBRs* in MFS 109. Stheneur *et al* demonstrated that the detection rate of *TGFBR1/2* mutant genes was 6.2% and 4.8% in classic MFS, and 6.2% and 4.6% in incomplete MFS out of 457 patients with MFS or related disorders 110. De Cario *et al* found 10 common polymorphisms of *TGFBR2* and 6 of *TGFBR1* in 75 patients with MFS. These polymorphisms were correlated with the severity of cardiovascular manifestations in MFS 111.

#### LTBP genes

LTBP1-4 are extracellular glycoproteins with a similar structure to fibrin. The complex of TGF-β1 and latency-related protein (LAP) is associated with LTBP through two disulfide bonds formed between the third 8-Cys domain of LTBP1, 3 and 4 and LAP, but not LTBP2 112. FBN1 microfibrils are responsible for the association between LTBP3 and 4 with the matrix, and the association between LTBP1 and matrix depends on a fibronectin network 113. The first, second and fourth 8-Cys domains of LTBP1 can independently bind to the matrix, and the N-terminal fragments of LTBP1 more readily bind to the ECM 114. The C-terminal LTBP1 fragment can form a bipartite interaction with a four-domain FBN1 fragment (EGF2-EGF3-Hyb1-cbEGF1), and LTBP1 connects with FBN1 through two independent epitopes, and have contacts with the ECM network through a flexible pivot 115.

*LTBP1* is located on chromosome 2p22.3 and has 38 exons. Quiñones-Pérez *et al* found a deletion of *LTBP1* in 3 patients with MFS in one family, and all patients had thoracic aortic aneurysm (TAA) and certain features of MFS, but they did not meet the criteria for MFS 116. Sticchi *et al* reported 5 mutations, including p.Asn542Ser and p.Lys2460Arg of *FBN1*, p.Val1739Met of *NOTCH1*, p.Arg1330Gln of *LTBP1*, and p.Arg423Trp of *TGFBR3* in an MFS patient with bicuspid aortic valve and aortic symptoms. These findings showed that the mutation of *LTBP1* may participate in regulating the vascular phenotype 117.

The *LTBP2* gene is located on 14q24.3 and consists of 36 exons. LTBP2 binds to the matrix through FBN and can negatively regulate the elastic fiber assembly through binding to fibulin-5. LTBP2 also regulates the activity of TGF-β signaling 118. LTBP2 is essential for the formation of microfibril bundles in the ciliary zonules 119. The mutation of *LTBP2* is related to EL and MFS 120, 121. *LTBP2* mutations may contribute to the systemic phenotype of syndromes related to the abnormality of genes that are associated with the TGF-β pathway 122. Ramona *et al* demonstrated that c.1642C>T (p.Arg548*) of *LTBP2* may contribute to ocular manifestations, MVP and pectus excavatum of MFS, but is not a causative gene 121.

*LTBP3* is mapped to 11q13.1, and is composed of 29 exons. In the *FBN1^mgR/mgR^: LTBP3^-/-^* mouse model, the lifespan of mice is prolonged compared with *FBN1^mgR/mgR^*. In mouse models with the absence of *FBN3*, it was observed that there was a decrease of activation of Smad2/3 and Erk1/2, reduction of disruption of aortic elastic fibers, and improvement of the circumferential mechanical properties of the thoracic aorta, whereas spinal deformities remained or were exacerbated, which may partly affect the overall aortic phenotype 123, 124. The improper localization of the FBN3/TGF-β complex may promote aortic disease. Bertoli-Avella *et al* reported that elastic fiber fragmentation, and collagen and proteoglycan deposition were increased in the aortic wall tissues of patients with p.Asp263His of LTBP*3*. They showed that the pathological manifestations in patients with this gene mutation included severe cardiovascular symptoms that were highly similar to MFS symptoms 125.

#### SKI

*SKI* is located on chromosome 1p36.33-p36.32 and contains 9 exons. This gene encodes the nuclear protooncogene protein homolog of avian sarcoma viral oncogene. It inhibits the phosphorylation of R-Smad through activated type I receptors, thereby restraining TGF-β-Smad signal transduction 126. *SKI* is associated with SGS, a systemic connective tissue disease that primarily manifests as cardiovascular, skeletal or craniofacial manifestations 127. Arnaud *et al* found that three different variants of the *SKI* gene affected the same amino acid (Thr180) in 6 patients with MFS or MFS-like/ Marfanoid syndrome and 3 patients with marfanoid habitus with learning disabilities. Additionally, 6 of the patients developed TAA. However, none of the patients had aortic dissection, and no aortic dilation (age-related) was found 128.

## The correlation of genotype/phenotype in MFS

The mutational heterogeneity of *FBN1* may cause age-related penetrance and diversification in clinical manifestations of MFS 129. Different mutations cause the sequestering of mutant protein in the endoplasmic reticulum then exerting haploinsufficiency (HI) efforts, or promoting secretion of mutant protein in ECM and exerting dominant-negative (DN) efforts. Pathogenic mechanisms and clinical manifestations of MFS may be related to the positional effects of mutations in *FBN1*, and dominance of the mutant alleles 130. In the DN model, *FBN1* mutant alleles interfere with the function of the protein encoded by the wild-type allele, thereby causing the appearance of MFS 131. And the HI model refers to the failure of microfibrillar assembly ascribed to HI of normal *FBN1* rather than the mutant protein 132. About one-third mutations of *FBN1* result in HI effects including nonsense, splicing or out-of-frame mutations, and other two-third mutations lead to DN effects including missense, splicing and in-frame mutations 133. DN Mutations of *FBN1* affects stability of microfibers on various conditions rather than the microfibril assembly, and therefore different pathological manifestations appear 134. Conceptually, HI mutations might have a consistent phenotype 135.

Some correlations of genotype/phenotype have been discussed. The widely accepted correlation is that large proportions of MFS children carry mutations located in exons 24-32 and in-frame mutations of *FBN1*, particularly in neonatal patients 48. And patients with mutations in exons 43-65 show the same high frequency of major cardiovascular phenotype compared with exons 24-32 136. Patients with mutations in exon 1-21 present with a high incidence of EL, and patients with mutations in exons 23-32 are more likely to develop aortic root dilatation 137. The more severe musculoskeletal and skin phenotype are observed in patients with an *FBN1* premature termination codon when compared with those in an in-frame mutation of *FBN1* 46, 138. MFS patients with truncating/splicing mutations have a higher proportion of aortic events and a younger median age, compared with MFS patients with missense mutations 90, 139. Mutations that alter the structure of the cbEGF module of FBN1 can affect the binding between FBN1 and calcium. Since FBN1 is more likely to be sensitive to proteolytic enzymes. Degradation of mutant fibers may occur during secretion or assembly in ECM 140. Patients with a missense mutation of *FBN1* involving a cysteine residue have a high risk of EL, and a mutation eliminating a cysteine shows a higher presence of aortic dilation and MVP than those with creating a cysteine 138. Moreover, Franken et al reported that MFS patients with HI variants had 2.5-fold increased risk for the combination of cardiovascular death and 1.6-fold increased risk for aortic complication compared with patients with DN variants after follow-up 8.2 years in 357 MFS patients 141.

Willis *et al* reported a 24-year-old patient with a de novo mutant c.3037G>A (p.G1013R) located in exon 25 of *FBN1.* The patient presented the cardiac involvement in early age and relative longevity. Even the mutant location is in exon 24-32 which is associated with neonatal MFS, the mutant G1013R of *FBN1* may be associated with a specific phenotype in the mutational hot spot 142. Whiteman* et al* reported two mutations in EGF domains of *FBN1* (C1117Y, C1129Y) may exert HI effect due to the protein product without being released from the endoplasmic reticulum 143. The classification of mutant types would influence the correlation of genotype-phenotype, assignment of the HI versus DN effects in MFS should base on the protein products and the effect of a specific mutation would be identified through experimental protein work 133, 135.

In addition, not only the diversity of *FBN1* mutations of genotype result in clinical heterogeneity, but also the effect of risk alleles in modifier genes and epigenetics are involved in thoracic aortic involvement 71, 144. Aubart *et al* found a second pathogenic event in aneurysm-related genes in nine MFS patients with disease-causing mutations of *FBN1* 71. They also identified two modifier regions containing *ECE1* and *PRKG1* respectively and a region closing to a cluster of MMPs by cross-mapping of genome-wide strategies in 1070 MFS patients with pathologic *FBN1* mutations, these regions were involved in the ECM regulation or SMCs relaxation hence contributing to aortic phenotype of MFS 71. Wu *et al* reported two MFS cases had two pathologic mutations of *FBN1* (c.A3142G/ c.G1622A; c.G1220A/c.C8080T), and the mutations in* PKD1* and *FBN1* were both found in 27 MFS patients without kidney disease 145. Furthermore, Arnaud *et al* found 4 probands carried missense homozygous and 5 probands with compound heterozygotes *FBN1* mutations in 2500 French MFS patients. All the patients were lack of the aggravated clinical manifestations 146. This report was contrary to previous reports, which demonstrated that homozygous and compound heterozygous cases presented with more severe symptoms compared with patients with only one mutation. These reports demonstrated that the diversification in clinical manifestations might result from a complex molecular mechanism.

## Epigenetic Regulation of Clinical Manifestations in MFS

Epigenetic regulation refers to the acquisition of new stable heritable traits that are not related to changes in the DNA sequence 147. Mechanisms of epigenetic regulation include DNA methylation, or modifications of DNA-associated histones and non-coding RNA 147. For MFS, molecular mechanisms of intrafamilial heterogeneity in the clinical severity remain unknown 148. It has been shown that epigenetics serves an essential role in regulation of aortic aneurysms in multiple studies. Arai *et al* found a negative correlation between DNA methylation on the WT allele within the *FBN1* CpG island shore and functional* FBN1* mRNA levels in human induced pluripotent stem cells, indicating that epigenetic regulation may affect *FBN1* expression in MFS 149. The increased activity of the methyltransferase EZH2 repressed SM22α, thus promoting aortic disorder in a* FBN1*^C1039G/+^ mouse model 150. Gomez *et al* demonstrated that an increase in histone H3 acetylation and methylation in the medial layer of TAA were associated with overexpression of *Smad2* signaling, specifically in SMCs over several passages in all types of TAA (caused by *FBN1* or *TGFBR2* mutations). This finding infers that heritability, the cell specificity and epigenetics may all contribute to the pathology of MFS 144. In a mouse model of *FBN1*^mgΔloxPneo^, 47.4% variation of the F2 skeletal phenotype could be explained by the potential modifier genes on chromosomes 3 and 6 participating in the TGF-β signaling pathway or other connective tissue diseases, whereas the modifier genes on chromosomes 4 and 13 were responsible for 40.7% variation of the F2 vascular phenotype 151.

## Abnormalities of TGF-β Pathway in MFS

**Figure 1:**
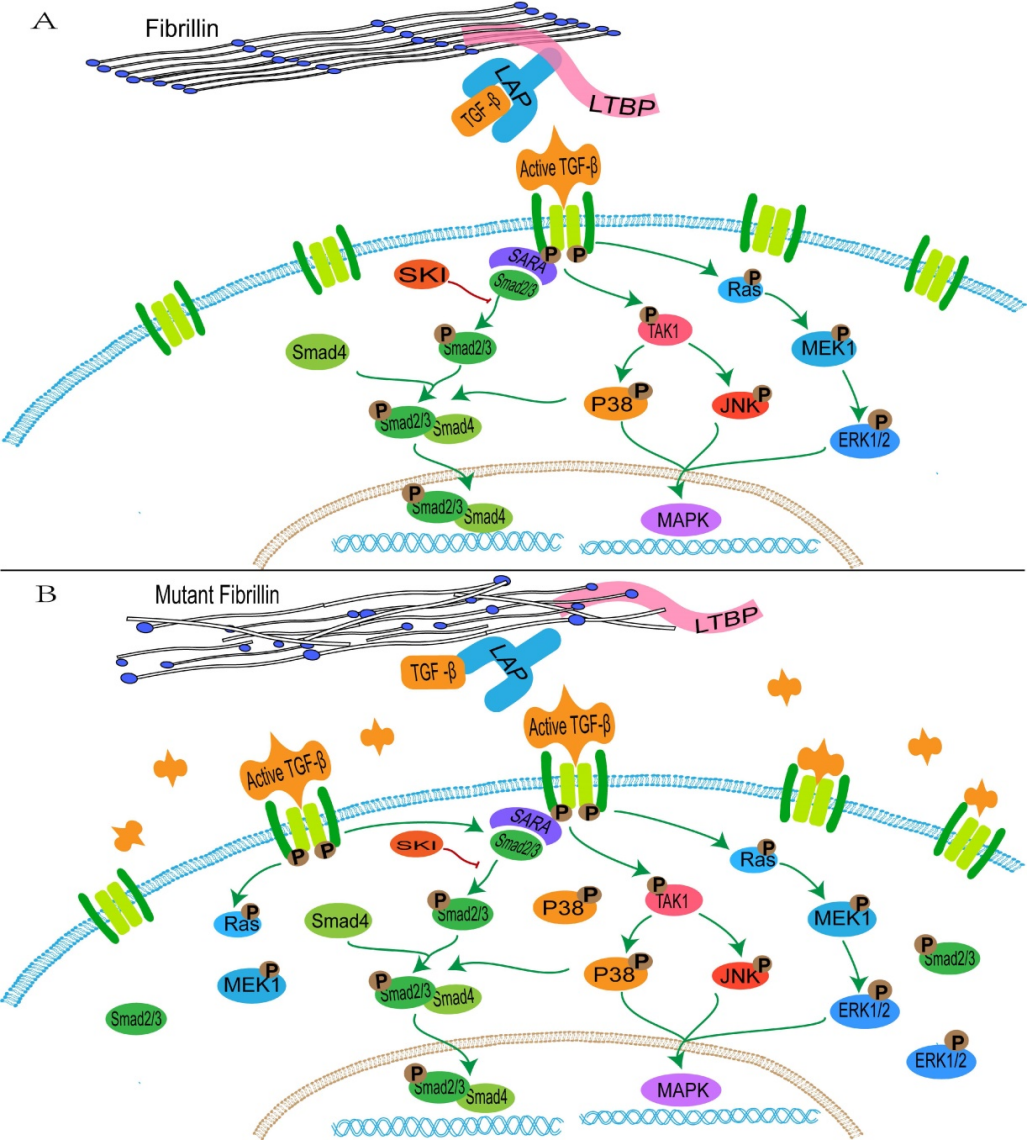
** The transduction of TGF-β pathway signaling**. A: the normal fibrillin-1 as a structural component of extracellular matrix microfibrils participates in the matrix sequestering of TGF-β. B: mutant fibrillin-1 loss interactions with LTBP, hence increasing the concentration of TGF-β in the extracellular matrix and upregulating the TGF-β pathway signal.

FBN1 regulates the concentration of activated TGF-β in the matrix through binding with the large latent complex, which consists of LAP, LTBP and TGF-β 152. Abnormalities of FBN1 lead to an imbalance in activation and signal transduction of TGF-β. Active TGF-β binds to the extracellular domain of the type I and type II receptor complex, and conformational changes of the intracellular serine/threonine domain of receptors promote phosphorylation and activation of type I receptors, thus resulting in activation of the SMAD and non-SMAD pathways (ERK, JNK and p38/MAPK) 153, 154 (Figure 1).

The activated TGFBR1 transmits signals through promoting the phosphorylation of a C-terminal Ser-Ser-X-Ser (SSXS) motif of SMAD2/3. SMAD4 forms a complex with active SMAD2/3 through the two phosphorylated serine residues at the C-terminal of SMAD2/3, and then SMAD4 is transferred to the nucleus 155. The N-terminal NH1 structures of SMAD3 and SMAD4 can bind to specific DNA sequences in the nucleus, whereas SMAD2 does not directly bind to DNA sequences 155. In addition, interactions between SMAD2/3 and the TGFBR1 is mediated by a Smad anchor for receptor activation (SARA), which interacts with SMAD2/3 through the Smad-binding domain and contacts with a TGFBR1 via a C-terminal region 156.

It is initially hypothesized that upregulation of the TGF-β pathway promotes the occurrence of TAA in patients with MFS. In the *FBN1*^C1039G/+^ mouse model, upregulation of Smad2, ERK1/2, MEK1 and p38 are observed in aortic tissues 157, 158, and the *Smad4* haploinsufficiency promotes activation of JNK1, which leads to aortic diseases 159. The selective inhibition of ERK1/2 reduces the pathological aortic root growth in an *FBN1*^C1039G/+^ mouse model 159. Conversely, in SMCs of patients with MFS, ERK activation drives the overexpression of Notch3, which may serve a protective role in aortic aneurysms through remodeling of tissues 160. The complex TGF-β signaling induces a mixed synthetic-contractile phenotype in SMCs, hence altering the normal physiological structure of the aorta 160. In addition, abnormal TGF-β signaling affects contractile protein and collagen 1 in vascular SMCs, thereby increasing cell stiffness and leading to aortic rigidity 161. TGF-β1 regulates the mitochondrial dynamics through the down-regulation of p-AMPK and induces the increase of reactive oxidative species (ROS) in the mitochondria in vascular progenitor cells of patients with MFS, excessive generation of ROS promotes the phosphorylation and translocation of p65-NF-κB from the cytoplasm to the nucleus in the vascular SMCs of patients with MFS, thus inducing cell senescence 162, 163.

It is worth noting that aortopathies have been observed in the absence of TGF-β signaling by deleting the *TGFBR2* in a young MFS mouse model (MFS-TBRII^-/-^ mice) 164. And this opposite conclusion is confirmed by other studies. Hu *et al* found that postnatal aortic homeostasis required physiologic TGF-β signaling, and disrupting SMCs TGF-β due to the loss of *TGFBR2* in postnatal MFS mouse model caused significant alterations of gene expression and severe aortic lesion including dilation, dissection, elastolysis, and etc. 165. Cook* et al* reported that the role of TGF-β was protective in the early stage of TAA formation and pathogenic in later stage in* Fbn1*^mgR/mgR^ mouse model 166.

Franken *et al* reported that angiotensin II (AngII) can directly induce Smad2 activation through AngII receptor-1(AT1r) and then increase TGF-β levels in an MFS mouse model, hence AngII may be the primary cause of aortic disease rather than TGF-β signaling, which may instead serve a role in secondary disease progression and appears to be a marker of aortic disease 167. Cook *et al* put forward an opposite result that due to the stage-specific dimorphic effects of TGF-β, TGF-β and AT1r were responsible for TAA progression through p-Erk1/2 and p-Smad2/3 signaling, respectively^166^. Moreover, systemic abrogated TGF-β activity in a C57BL/6 mouse model treated with anti-TGF-β antibodies promotes the formation of Ang II-induced abdominal aortic aneurysms (AAAs) 168. The contradictory results between MFS and AAAs may be explained by the difference in pathological characteristics. Increased TGF-β signaling promotes non-inflammatory excessive accumulation of SMCs and ECM in MFS, and other AAAs are related to the thinned arterial walls, loss of SMCs and ECM, and vascular inflammation 168.

Additionally, androgens enhance Erk/Smad activation, as elastic fiber fragmentation and matrix metalloproteinases 2 (MMP2) expression are higher in males compared with females in MFS mice 13, 169. MMP2 cleaves matrix proteins in medial SMCs of MFS and can regulate the levels of TGF-β and Erk1/2 phosphorylation 170.

## Conclusion

MFS is a complex disease involving multiple systems. The clinical manifestations of MFS are varied and overlap with other diseases. The clinical symptoms of MFS are gradually aggravated with age, and EOMS cases always have serious clinical outcomes. Diagnosis of MFS relies on family history and multi-system scores. Due to the clinical heterogeneity and diversification of mutations, it is difficult for doctors to diagnose mild cases or prenatal patients 171. However, with the advancements in genetic testing, this problem is expected to be solved.

The majority of the *FBN1* mutations in the MFS family affect a single amino acid and lead to abnormalities in protein function. Pathogenic mechanisms of *FBN1* include the HI and DN model. But neither model is sufficient to explain the heterogeneity of clinical manifestations. The epigenetic regulation only explains part of the clinical differences. In addition, other genes associated with related disease have been discovered over the years, such as *TGFBRs, LTBP2, LTBP3* and *SKI*. Nevertheless, mutations of these genes are also detected in other diseases, and symptoms caused by them may not be as severe as those caused by *FBN1* mutations. Further *in vitro*/*in vivo* studies are required to identify the pathogenesis of these genes, the effects of their interactions and the genotype-phenotype correlations.

Abnormalities of the TGF-β pathway signaling is another important pathological mechanism by which aneurysms develop in patients with MFS. It is specifically observed in the SMCs of the vascular medial layer. The TGF-β pathway can regulate a number of processes in several types of cells, including endothelial cells, SMCs and pericytes, amongst others, and its mechanisms may be associated with the induction of proliferation, apoptosis, migration, adhesion, ECM protein production and cytoskeletal organization 172. The up- and down-regulation of TGF-β pathway signaling are both related to MFS, canonical and non-canonical signaling exert antagonistic effects and co-adjust the mechanism of development of aneurysms in MFS. A better understanding of the TGF-β pathway signaling may assist in determining the exact molecular mechanism underlying the development of MFS. At present, the role of TGF-β pathway signaling abnormalities in MFS requires further elucidation.

With the development of next-generation sequencing technologies, WES is being widely used to detect genes related to MFS. In addition, research methods based on the single-cell analysis, such as scRNA-seq, have also been used for analysis of the expression of genes in various tissues, and explaining the differing degrees of involvement of each system. Along with whole gene technology and bioinformatics, the combined application of high throughput sequencing technologies and 'big data' analysis will be helpful in further analyzing the entire genome and the better understanding of the complex traits of patients with MFS. This may highlight novel strategies for prevention, early diagnosis and treatment/management of MFS.

## Figures and Tables

**Table 1:** Genes associated with MFS and related diseases

Gene	Chromosomal region	Functions	Involvement in disease	Roles in TGF-β pathway	Reference
FBN1	15q21.1	A component of calcium-binding microfibrils, provide force-bearing structural support in connective tissue	MFS, MASS, EL, WMS, SGS, NPS	Maintain matrix structure and function, participate in the matrix sequestering of TGF-β	91-93
FBN2	5q23.3	A component of connective tissue microfibrils, assembled into elastic fiber	CCA	Maintain matrix structure and function, participate in the matrix sequestering of TGF-β	98-100
TGFBR1	9q22.33	Transduce the TGF-β signal	LDS	Transduce of TGF-β pathway signaling from the cell surface to the cytoplasm	101-103, 109
TGFBR2	3p24.1	Phosphorylates proteins and regulates the transcription of genes	MFS, LDS, tumors	Transduce of TGF-β pathway signaling from the cell surface to the cytoplasm	101-103, 109
LTBP-1	2p22.3	Targets the TGF-β to extracellular matrix	N/A	Regulate the concentration of TGF-β	112-115
LTBP-2	14q24.3	A component of TGF-β latent complex, a structural component of microfibrils	PCG, MSPKA, WMS3	Regulate the concentration of TGF-β	118,119
LTBP-3	11q13.1	Combines with TGF-β, a structural component of extracellular matrix	DASS, GPHYSD3	Regulate the concentration of TGF-β	112,113
SKI	1p36.33-p36.32	A repressor of TGF-β signaling, regulates the neural tube development and muscle differentiation	SGS	Inhibit the phosphorylation of Smad2 and Smad3	126,127

Abbreviations: CCA, Congenital Contractural Arachnodactyly; DASS, Dental anomalies and short stature; EL, ectopia lentis syndrome; GPHYSD3, Geleophysic dysplasia 3; LDS, Loeys-Dietz syndrome; MASS, Mitral valve, Aorta, Skeleton, Skin involvement; MFS, Marfan syndrome; MSPKA, Microspherophakia; NPS, Neonatal Progeroid syndrome; PCG, Primary Congenital Glaucoma; SGS: Shprintzen-Goldberg syndrome; WMS, Weill-Marchesani syndrome; N/A, not applicable.
